# Therapeutic Potential of Stem Cells from Human Exfoliated Deciduous Teeth in Models of Acute Kidney Injury

**DOI:** 10.1371/journal.pone.0140121

**Published:** 2015-10-28

**Authors:** Yuka Hattori, Hangsoo Kim, Naotake Tsuboi, Akihito Yamamoto, Shinichi Akiyama, Yiqin Shi, Takayuki Katsuno, Tomoki Kosugi, Minoru Ueda, Seiichi Matsuo, Shoichi Maruyama

**Affiliations:** 1 Department of Oral and Maxillofacial Surgery, Nagoya University Graduate School of Medicine, 65 Tsurumai-cho, Showa-ku, Nagoya, Aichi, Japan; 2 Department of Nephrology, Internal Medicine, Nagoya University Graduate School of Medicine, 65 Tsurumai-cho, Showa-ku, Nagoya, Aichi, Japan; University of Torino, ITALY

## Abstract

**Background:**

Acute kidney injury (AKI) is a critical condition associated with high mortality. However, the available treatments for AKI are limited. Stem cells from human exfoliated deciduous teeth (SHED) have recently gained attention as a novel source of stem cells. The purpose of this study was to clarify whether SHED have a therapeutic effect on AKI induced by ischemia-reperfusion injury.

**Methods:**

The left renal artery and vein of the mice were clamped for 20 min to induce ischemia. SHED, bone marrow derived mesenchymal stem cells (BMMSC) or phosphate-buffered saline (control) were administered into the subrenal capsule. To confirm the potency of SHED *in vitro*, H_2_O_2_ stimulation assays and scratch assays were performed.

**Results:**

The serum creatinine and blood urea nitrogen levels of the SHED group were significantly lower than those of the control group, while BMMSC showed no therapeutic effect. Infiltration of macrophages and neutrophils in the kidney was significantly attenuated in mice treated with SHED. Cytokine levels (MIP-2, IL-1β, and MCP-1) in mice kidneys were significantly reduced in the SHED group. In *in vitro* experiments, SHED significantly decreased MCP-1 secretion in tubular epithelial cells (TEC) stimulated with H_2_O_2_. In addition, SHED promoted wound healing in the scratch assays, which was blunted by anti-HGF antibodies.

**Discussion:**

SHED attenuated the levels of inflammatory cytokines and improved kidney function in AKI induced by IRI. SHED secreted factors reduced MCP-1 and increased HGF expression, which promoted wound healing. These results suggest that SHED might provide a novel stem cell resource, which can be applied for the treatment of ischemic kidney injury.

## Introduction

Acute kidney injury (AKI) is a critical condition associated with high mortality rates of 30 to 50%. AKI has various pathoetiologies; ischemic kidney diseases are the main cause of AKI and ischemia-reperfusion injury (IRI) of the kidney is one of the key AKI models. AKI can result in remote organ dysfunction involving the heart, lungs, liver, intestines, and brain [1]. AKI contributes to the long-term effects of injury, such as interstitial fibrosis [2–5]. The pathophysiology of IRI injury is multifactorial, and depends on a number of factors, including endothelial injury of peritubular small vessels, immune responses, inflammatory processes, and necrosis and apoptosis of the renal tubular epithelium [6]. However, the molecular, cellular, and genetic mechanisms underlying these effects remain largely unknown, and there is no effective therapy for ischemic kidney diseases.

Preclinical studies suggest that administered mesenchymal stem cells (MSC) ameliorate renal injury and promote kidney repair [7, 8]. MSC migrate to damaged kidney tissue and secrete several cytokines and chemokines that can alter the course of injury. MSC are thought to regulate the immune response resulting in tissue repair and remodeling through paracrine and/or endocrine mechanisms. MSC exist in virtually every tissue of the human body and exert therapeutic effect in various diseases. Bone marrow is a major source of MSC, however, the number of bone marrow–derived mesenchymal stem cells (BMMSC) and their potential for proliferation and differentiation declines with increasing age. In addition, bone marrow aspiration is an invasive procedure for donors [9].

Stem cells from human exfoliated deciduous teeth (SHED), which are considered medical waste, have gained attention recently as a novel source of stem cells. SHED can be isolated from human dental pulp and expanded easily. Similar to BMMSC, SHED are capable of extensive proliferation and multipotential differentiation [10–12]. The advantage of SHED compared with BMMSC is that they can be obtained noninvasively and possess a highly proliferative capacity and advanced interactivity with biomaterials used for tissue engineering applications[13]. Furthermore, microarray analysis indicates that SHED exhibit higher expression levels of several growth factors, including fibroblast growth factor, transforming growth factor, connective tissue growth factor, nerve growth factor, and bone morphogenetic protein [14]. These data demonstrate that SHED could offer a more beneficial and useful cell source than BMMSC. Preclinical studies have demonstrated the therapeutic effects of SHED in various models including bone defect, skin ulcer, cord injury, and neonatal hypoxia-ischemia [15–18]. The purpose of this study was to determine whether SHED show a therapeutic effect on IRI-induced AKI.

## Materials and Methods

### Animals

Animal experiments were performed in accordance with the Animal Experimentation Guidelines of Nagoya University Graduate School of Medicine. Seven to 8-week-old male C57BL/6j mice weighing 18–22 g obtained from the Chubu Kagaku Shizai Corporation (Nagoya, Japan), were used. Animals were housed at a constant temperature and humidity, with a 12:12-h light-dark cycle, and had unrestricted access to a standard diet and tap water in accordance with the National Institutes of Health Guide for the Care and Use of Laboratory Animals. The experimental protocols were approved by the Animal Experiment Committee of Nagoya University Graduate School of Medicine (Permit Number: 27206). All surgery was performed under with pentobarbital and diethyl ether, and all efforts were made to minimize suffering.

### Isolation of stem cells from human exfoliated deciduous teeth (SHED) and cell culturing

SHED were isolated as previously described [11, 19]. Briefly, exfoliated deciduous teeth (from 6–12 year old individuals) were collected. Experimental protocols were approved by the ethics committee of Nagoya University Graduate School of Medicine according to the Declaration of Helsinki (Permit Number: bio12). Written informed consent was provided from each patients’ guardians and informed assent was obtained from children. Subsequent to separation of the crown and root, the dental pulp was isolated and then digested in a solution of 3 mg/ml collagenase type I (Worthington Biochem, Freehold, NJ, USA) and 4 mg/ml dispase (Roche Diagnostic/Boehringer Mannheim Corp., Indianapolis, IN, USA) for 1 h at 37°C. Single-cell suspensions (1 x 10^4^ to 2 x 10^4^ cells/ml) were plated on culture dishes containing Dulbecco's modified Eagle medium (DMEM, Sigma-Aldrich, Tokyo, Japan) supplemented with 10% fetal calf serum (FCS, Sigma-Aldrich, Tokyo, Japan) and were then incubated at 37°C in 5% CO_2_. These techniques resulted in a cell population that we have termed SHED. SHED were characterized as expressing a set of mesenchymal stem cell markers (i.e., CD90, CD73,CD105 and CD44), but not endothelial/ hematopoietic markers (i.e., CD45, CD34, CD11b,CD14 and HLA-DR) using flow cytometry analysis [17, 20]. Similar to BMMSC, SHED exhibited adipogenic, chondrogenic, and osteogenic differentiation as previously described [11, 19].

### Preparation of BMMSC and skin-fibroblast(FB)

Human BMMSC were purchased from Lonza (26737, Tokyo, Japan), and were cultured on culture dishes containing DMEM supplemented with 10% FCS at 37°C in 5% CO_2_. Human skin-fibroblasts (FB) purchased from the Health Science Research Resources Bank (TIG-114, Tokyo, Japan) were also cultured in DMEM supplemented with 10% FCS.

### Conditioned medium from SHED (SHED-CM)

Conditioned medium from stem cells of human exfoliated deciduous teeth (SHED-CM) was generated as follows: SHED (passage 7–9) were seeded at 1 x 10^6^ cells/10 ml/10-cm tissue culture dish for 24 h at 37°C in 5% CO_2_. Next, the conditioned medium in each dish was removed and washed twice with phosphate-buffered saline (PBS) and twice with serum-free media to remove cell debris. Subsequently, SHED were cultured in serum-free media for 48 h. The SHED-CM was collected by centrifugation at 1500 rpm for 5 min, followed by centrifugation at 3000 rpm for 5 min to remove cell debris.

### In vivo kidney ischemia reperfusion injury experiments

#### Animal model for monolateral kidney IRI and cell transplantation

Mice were underwent right heminephrectomy under anesthesia with pentobarbital and diethyl ether. Mice were maintained at 39°C during surgery, and fluid loss during surgery was replaced with 1.0 ml saline. Seven days post-surgery, the left renal artery and vein of the C57BL/6 mice were clamped to induce ischemia. Twenty minutes later, the clamp was released and allowed to reperfuse (day 0). Once kidney color had been confirmed as having reverted back to red, SHED (passage 7–9, 1 x 10^6^ cells/10 μl PBS) or human BMMSC (passage 5, 1 x 10^6^ cells/10 μl PBS) or 10 μl PBS (as control) were administered into the subrenal capsule. Tissue and blood samples were collected at various time points.

#### Renal Function

Blood samples were collected at each time point to evaluate renal function, and blood urea nitrogen (BUN) and serum creatinine (sCr) levels were measured by Mitsubishi Chemical Medience Co. Ltd (Tokyo, Japan).

#### Histology and tubular injury score

Kidney samples were processed for routine histology as previously described [21]. For light microscopy analysis, kidneys were embedded in paraffin. Serial sections (1μm thick) were obtained for conventional histological assessment such as hematoxylin and eosin (H&E) or periodic acid-Schiff (PAS) staining. Histological changes due to acute tubular necrosis (ATN) were evaluated in the outer medulla in PAS-stained tissue and were quantified by counting the percent of tubules that displayed cell necrosis, loss of brush border, cast formation, and tubule dilatation and scored as follows: 0, none; 1, <10%; 2, 11–25%; 3, 26–45%; 4, 46–75%; and 5, >76% [22].

#### SHED tracking assay

To evaluate the tracking of administered SHED, kidney samples were collected on days 2, 7, and 14. Paraffin sections (1μm thick) were incubated with rabbit anti-human Lamin A/C antibody (EPR4100; Abcam plc, Cambridge, UK) subsequent to blocking with 10% normal goat serum (Jackson Immuno-Research Laboratories, Inc., USA) for 30 min. The primary antibody was visualized using the Histofine Simple Stain PO(R) kit (Nichirei, Tokyo, Japan) according to the manufacturer’s instructions. Slides were counterstained with hematoxylin [23]. Tissue images were taken with a Zeiss Z1 microscope and Axiovision Windows software version 4.4 (Carl Zeiss, Oberkochen, Germany).

#### Immunohistochemistry

Kidney tissue samples were embedded in optimal cutting temperature (OCT) compound and frozen in liquid nitrogen for immunofluorescent staining. Immunofluorescence was performed on cryosections (3-μm thick). For indirect immunofluorescence, nonspecific binding sites were blocked with 10% normal goat serum for 30 min. The cryosections were stained with primary antibodies: rat anti-mouse Ly-6B (clone MCA771G, AbD Serotec, Kidlington, UK) or rat anti-mouse F4/80 (clone MCA497G, AbD Serotec, Kidlington, UK) or rabbit anti-human CD3 (clone SP7, Spring Bioscience Inc., USA). For fluorescent visualization of bound primary antibodies, sections were incubated with FITC-conjugated secondary antibodies; goat F(ab')2 anti-rat IgG (clone STAR69, AbD Serotec, Kidlington, UK) or goat anti-rabbit IgG (113-095-144, Jackson Immuno Research Laboratories, Inc., USA) for 30 min. For the negative controls, the staining procedure was performed with rat IgG2a (clone RTK2758, BioLegend Inc., USA) or rat IgG2b (clone RTK4530, BioLegend Inc., USA) or rabbit IgG. To detect proliferating cells, paraffin sections (1μm thick) were incubated with anti Ki67 antibody (SP6; abcam discover more, Tokyo, Japan) for 60min subsequent to blocking with 10% normal goat serum (Jackson Immuno-Research Laboratories, Inc., USA) for 30 min. The primary antibody was visualized using the Histofine Simple Stain PO(R) kit (Nichirei, Tokyo, Japan) according to the manufacturer’s instructions. Slides were counterstained with hematoxylin. All incubations were performed in a humid chamber at room temperature. Tissue images were taken with a universal fluorescence microscope (BZ9000, Keyence Osaka, Japan). Infiltrating Ly6B-positive cells, F4/80-positive cells and Ki67-positove were quantitated in the outer medulla of the injured kidney. Semiquantitative analysis of cell infiltration was done by counting the number of positively labeled cells in 10 (Ly-6B neutrophils original magnification, x200) or 20 (F4/80 macrophages, original magnification, x400) or 20 (CD3 T cells, original magnification, x400) non-overlapping HPFs.

#### Measurement of cytokine and chemokine levels

Kidney samples were homogenized in Tissue Protein Extraction Reagent (T-PER, Pierce Biotechnology, Inc., USA) centrifuged at 10,000x*g* for 5 min at 4°C, and the supernatants were collected. Macrophage inflammatory protein (MIP-2), interleukin-1βterleβ), and monocyte chemotactic protein-1 (MCP-1) concentrations in the kidney were determined by sandwich enzyme-linked immunosorbent assay (ELISA; R&D Systems, Inc., USA&Canada). Total protein concentrations were determined using the bicinchoninic acid protein assay kit (Pierce Biotechnology, Inc., USA). The results were normalized to the total protein concentration.

### In vitro experiments with tubular epithelial cells (TEC)

#### Isolation of TEC and cell culturing

Tubular epithelial cells (TEC) were isolated from the renal cortex of C57BL/6 mice followed by brief collagenase digestion. Cells were plated on collagen coated dishes (Asahi Glass Co, Tokyo, Japan) in K1 medium (224.25ml Ham’s F12, 226.25 ml DMEM, and 12.5 ml HEPES) containing 10% fetal bovine serum, hormones (epidermal growth factor [50 pg/ml]; insulin-transferrin-sodium selenite media supplement [0.12 IU/ml]; prostaglandin E1 [1.25 ng/ml]; hydrocortisone [18ng/ml]; T3 [34 pg/ml]), and 1% penicillin/streptomycin (Invitrogen-Gibco, Co.,USA) then incubated at 37°C in 5% CO_2_, as previously described [24, 25]. Cells were used between the fourth and fifth passage. Expression of epithelial cell markers including mouse anti-pan cytokeratin (clone PCK-26, Sigma-Aldrich, Tokyo, Japan) was verified by immunofluorescence assays on subconfluent monolayers, (data not shown), as previously described [26, 27].

#### H_2_O_2_ stimulation assay

TEC and vascular endothelial cells (RCB1994, Riken BRC, Saitama, Japan) were seeded at 5 x 10^4^ cells/1 ml/well in 24-well collagen coated plates (Asahi Glass Co, Tokyo, Japan) for 24 h. Subsequently, the supernatant in each well was removed and cultured in either Control [1 ml DMEM with 0.4 mM hydrogen peroxide (H_2_O_2_)] or SHED-CM (1 ml conditioned medium from SHED with 0.4 mM H_2_O_2_]. MCP-1 protein concentrations in the culture supernatants were determined at each time point by quantitative sandwich ELISA (R&D Systems, Inc., USA&Canada). Assays were performed in triplicate and experiments were repeated five times.

#### Scratch wound assays with TEC

The paracrine effect of SHED was studied using an *in vitro* wound healing assay as previously described [28, 29]. TEC were cultured on 35-mm glass base dishes (Asahi Glass Co, Tokyo, Japan) as confluent monolayers. These confluent monolayers were then scraped with a 200-ml sterile pipette tip. The role of hepatocyte growth factor (HGF) in SHED-CM was examined by means of anti-human HGF antibody (R&D Systems, Inc., USA&Canada). As for this antibody, the IgG fraction at a concentration of 10 μg/mL was able to neutralize a biological activity of 100 ng/mL HGF. Normal goat IgG fraction (10 μg/mL) was employed as a control. These neutralizing antibodies or control were added to conditioned media, and assay was performed. The wounded monolayer was washed three times with serum-free media to remove cell debris and replaced with 2 ml/dish SHED-CM, SHED-CM with anti HGF antibody, SHED-CM with normal goat IgG or control medium (DMEM). Wound closure was monitored and images were collected at various time points subsequent to scratching until closure was either complete or no longer progressing. Optical images were captured with an inverted microscope (IX70, Olympus, Tokyo, Japan) and digital camera (C-5060, Olympus, Tokyo, Japan), and analyzed with ImageJ software (National Institutes of Health, Bethesda, MD, USA). The wound-healing effect was calculated as a percentage of the remaining cell-free area compared with the area at the start of the experiment. Each assay was performed in triplicate and experiments were repeated five times.

#### Measurement of HGF expression in SHED, BMMSC, and FB

SHED at passage 7–9, human BMMSC at passage 5, and FB at passage 5 were used. SHED, BMMSC, and FB were cultured in DMEM and samples were collected at 48 h. The conditioned media from BMMSC, FB, and SHED were analyzed using HGF ELISA (R&D Systems, Inc., USA & Canada). Data are expressed as picograms of the secreted factor per 10^6^ cells at the time of harvest. Each assay was performed in triplicate and experiments were repeated three times.

### Statistical analysis

Data are expressed as calculated mean ± standard deviation (mean ± SD). Statistical analysis was performed using the unpaired, two-tailed Student’s *t*-test for single comparisons using Excel software (2010, Microsoft, USA). For multiple comparisons, SPSS 22.0 statistical software (SPSS Inc, Chicago, IL) was used. Statistical significance was evaluated by ANOVA to determine the significance of differences between experimental groups. When a statistically significant difference was indicated by ANOVA, further analysis was performed using Tukey’s test to determine the significance of differences between any pair of groups. *P* < 0.05 was regarded as statistically significant.

## Results

### In vivo studies

#### Renal function

**Fig 1:**
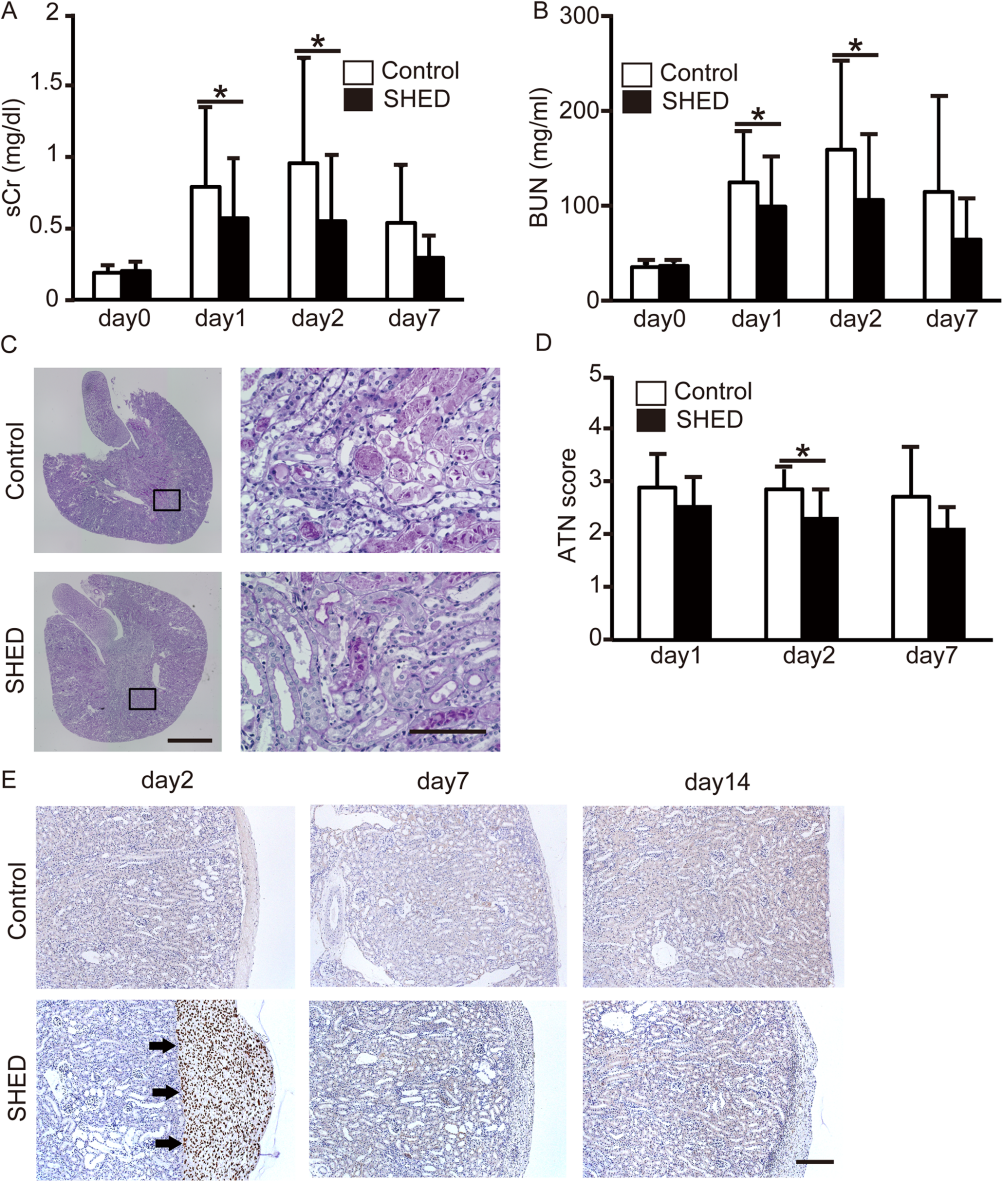
The effects of stem cells from human exfoliated deciduous teeth (SHED) transplantation, renal function, and histology. (A, B) Renal function as determined by serum creatinine (sCr) and blood urea nitrogen (BUN) levels. Values are presented as mean ±SD; SHED group, day0 (n = 36), day1 (n = 36), day2 (n = 24), day7 (n = 16); control group, day0 (n = 36), day1 (n = 36), day2 (n = 23), day 7 (n = 14).* Significant difference (*P*<0.05 vs. control). (C, D) Renal tissue pathology in each group (PAS staining). The acute tubular necrosis (ATN) score was evaluated in the outer medulla. (C) Representative PAS–stained sections of outer medulla on day2 from control and SHED treated animals (original magnification, left side panels; x4 Scale bar: 1 mm, right side panels; x400 Scale bar: 100 μmm). (D) Histograms of SHED ATN scores. Values are presented as mean ±SD; SHED group, day1 (n = 9), day2 (n = 8), day7 (n = 8); control group, day1 (n = 9), day2 (n = 7), day 7 (n = 7). (E) Representative micrographs of administered SHED in subrenal capsule using anti-human lamin A/C (arrows, original magnification, x100 Scale bar: 200 μmm). SHED group, day2 (n = 4), day7 (n = 4), day14 (n = 4); control group, day2 (n = 4), day7 (n = 4), day 14 (n = 4).* Significant difference (*P*<0.05 vs. control).

**Fig 2:**
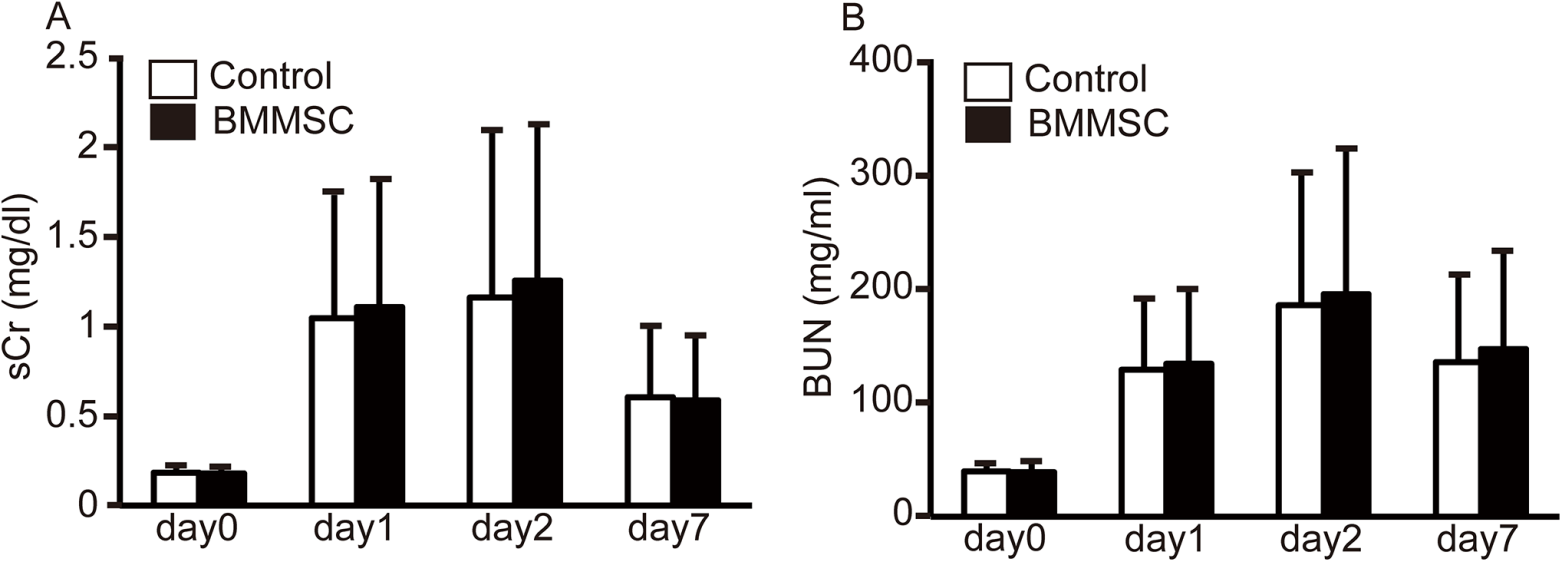
The effects of Bone marrow–derived mesenchymal stem cells (BMMSC) transplantation, renal function. (A, B) Renal function as determined by serum creatinine (sCr) and blood urea nitrogen (BUN) levels. Values are presented as mean ±SD; BMMSC group, day0 (n = 35), day1 (n = 35), day2 (n = 35), day7 (n = 7); control group, day0 (n = 34), day1 (n = 34), day2 (n = 34), day7 (n = 7).

The levels of sCr and BUN increased post IRI operation and both peaked at day 2 post-ischemia. Renal function had been almost normalized by day 7. sCr and BUN levels on days 1 and 2 were significantly lower in the SHED group than in the control group (Fig 1A and 1B). Renal damage was detected predominantly in the outer medulla manifesting as cell necrosis, loss of brush border, cast formation, and tubule dilatation. Tubular damage was more severe in the control group than in the SHED group on day 2; the SHED group ATN score on day 2 was significantly lower (Fig 1C and 1D). Nevertheless, there was no significant difference in the levels of sCr and BUN of the BMMSC group compared with the control group (Fig 2).

#### Tracking of administered SHED or BMMSC

To investigate the localization of the administered SHED or BMMSC or control, human lamin A/C immunostaining was performed on day 2, 7, and 14. This antibody reacts with human, but not with mouse or rat [30–33]. SHED were observed at the site of injection in the subrenal capsule on day 2; no migration into renal tissue was observed (Fig 1E). On the other hand, BMMSC were observed on day2 and 7 (S1A Fig). Both SHED and BMMCS induced inflammatory cell infiltration at the cell injected site of the renal subrenal capsule on days 7 and 14(S1B and S1C Fig). Immunostaining showed that most of the infiltrated cells were T cells (S2 Fig, S3 Fig).

#### Inflammatory cell infiltration in the kidneys

**Fig 3:**
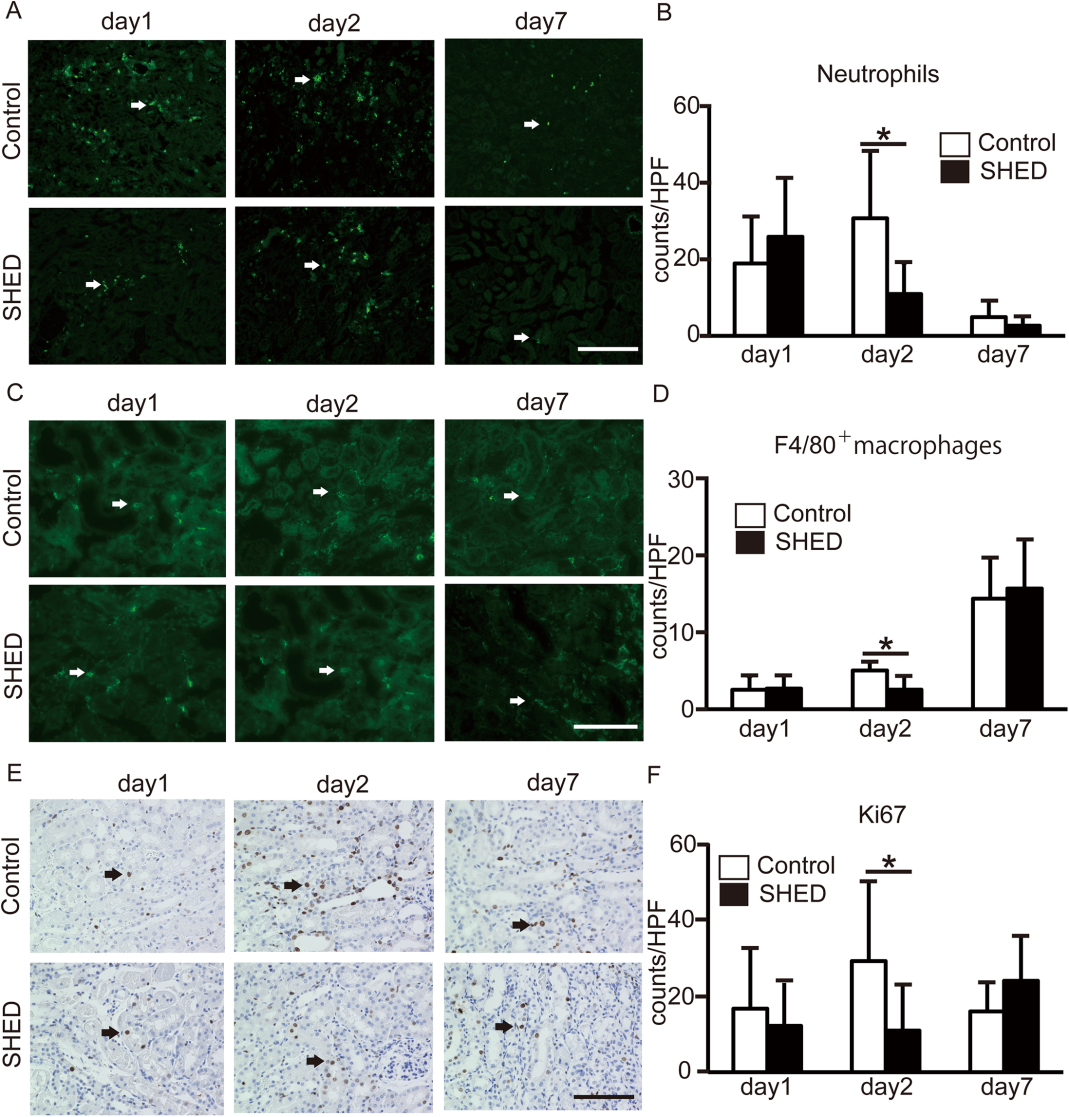
Immunofluorescence staining of neutrophils, macrophages and proliferating cells in renal tissues. The number of infiltrating neutrophils or macrophages in the control and SHED groups was assessed by immunofluorescence staining in 10 or 20 non-overlapping view fields of the outer medulla. (A) Representative immunofluorescence staining of neutrophils using anti-Ly-6B (arrows, original magnification, x200 Scale bar: 200 μmm). (B) Histograms of the infiltration of Ly-6B positive cells. (C) Representative immunofluorescence staining of macrophages using anti-F4/80 (arrows, original magnification, x400 Scale bar: 100 μmm). (D) Histograms of the infiltration of F4/80 positive cells. (E) Representative immunofluorescence staining of proliferating cells using anti-Ki67 (arrows, original magnification, x400 Scale bar: 100 μmm). (F) Histograms of the infiltration of Ki67 positive cells. Values are presented as mean ±SD; SHED group, day1 (n = 9), day2 (n = 8), day7 (n = 8); control group, day1 (n = 9), day2 (n = 7), day 7 (n = 7).* Significant difference (*P*<0.05 vs. control).

Inflammatory cell infiltration was studied by F4/80 and Ly-6B immunohistochemistry. Control staining using rat IgG2a, rat IgG2b or rabbit IgG were performed to give a sense of background for tissue (S4 Fig). Pathologically, inflammatory cells such as neutrophils and macrophages were found to have mainly infiltrated the outer medulla. The number of neutrophils reached its peak on day 2 and macrophage levels were increased at day 7 post-ischemia. The number of neutrophils and macrophages was significantly reduced in the SHED group on day 2 (Fig 3).

#### Proliferating cells in the kidneys

Proliferating cells were studied by Ki67. When AKI is induced, the number of proliferating tubular cells increases[2]. In our study, proliferating tubular cells were mainly detected at the outer medulla on days 1, 2 and 7 post IRI. The number of proliferating cell was significantly reduced in the SHED group on day 2 (Fig 3E and 3F).

#### Cytokine and chemokine expression in the kidneys

**Fig 4:**
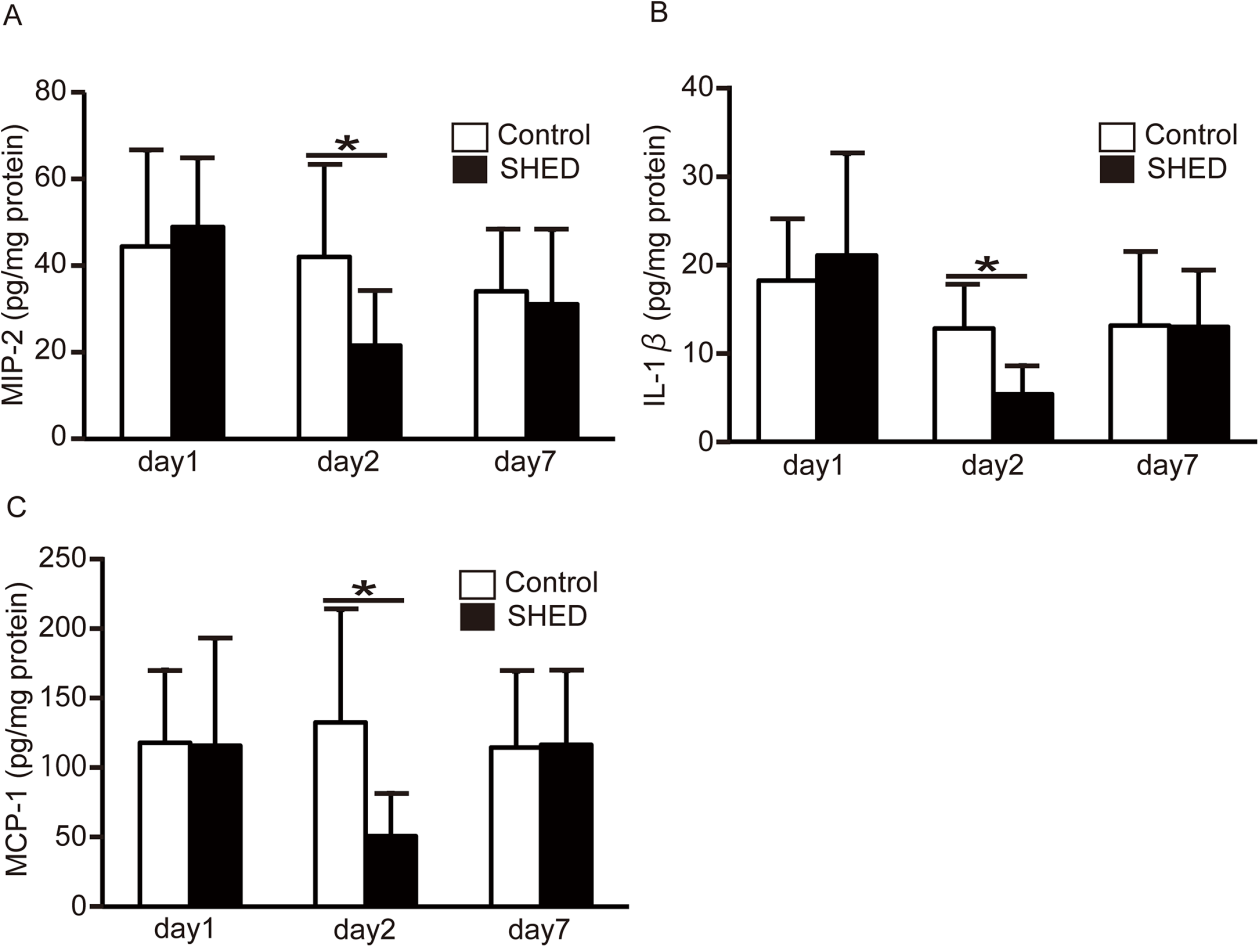
Cytokine and chemokine expression in the kidney. The protein levels of MIP-2 (A), IL-1β (B), and MCP-1 (C) were determined by quantitative sandwich ELISA and corrected for the total amount of protein. Values are presented as mean ±SD; SHED group, day1 (n = 9), day2 (n = 8), day7 (n = 8); control group, day1 (n = 9), day2 (n = 7), day 7 (n = 7).* Significant difference (*P*<0.05 vs. control).

Kidney cytokines and chemokines were quantitatively evaluated by ELISA. Expression of MCP-1, MIP-2, and IL-1β was not detected in normal kidneys (data not shown), but was detected in injured kidneys. MCP-1, MIP-2, and IL-1β protein levels were significantly lower in the SHED group than in the control group on day 2 (Fig 4).

### In vitro studies

#### Characterization of SHED

Flow cytometry analysis showed that SHED expressed MSC markers (CD90, CD73, CD105 and CD44), but not endothelial/hematopoietic markers (CD45, CD34, CD11b, and HLA-DR) (Table 1).

**Table 1:** Flow cytometry analysis of SHED.

	SHED (N = 4)	
**MSC markers**	**Positive (%)**	**SD**
CD90	98.45	0.25
CD73	99.95	0.06
CD105	97.58	2.01
CD44	99.90	0.08
**Negative markers**		
CD45	0.20	0.06
CD34	0.30	0.13
CD11b	0.26	0.13
CD14	0.21	0.10
HLA-DR	0.29	0.07

Values are presented as mean ±SD; (n = 4).

#### Cytokine and chemokine expression in tubular epithelial and vascular endothelial cells

**Fig 5:**
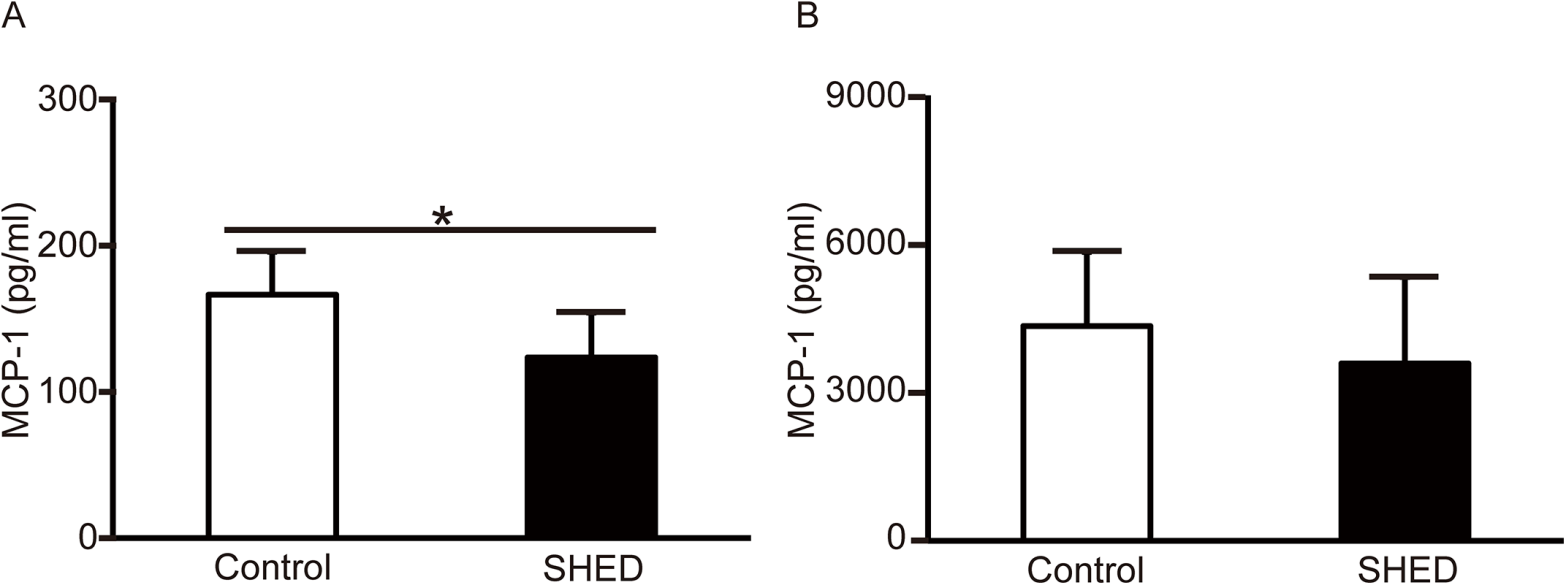
MCP-1 expression subsequent to stimulation with 0.4 mM H_2_O_2_. The expression of MCP-1 protein was determined by quantitative sandwich ELISA at 24 h. (A) The level of MCP-1 secreted from TEC. (B) The level of MCP-1 secreted from vascular endothelial cells. Each assay was performed in triplicate, and experiments were repeated five times. Values are presented as mean ±SD; SHED group (n = 5); control group (n = 5).* Significant difference (*P*<0.05 vs. control).

Conditioned media were collected subsequent to culturing with SHED for 48 h. In order to determine the SHED mechanism of activity, the effect of secreted factors was examined. SHED-CM reduced MCP-1 protein concentrations at 24 h in the conditioned media of cultured TEC stimulated with H_2_O_2_, indicating that SHED secreted factors reduce MCP-1. However there was no significant difference in MCP-1 protein concentrations at 24 h in the conditioned media of cultured vascular endothelial cells (Fig 5). MIP-2 and IL-1β proteins were not detected in the conditioned media (data not shown).

#### Scratch wound assays

**Fig 6:**
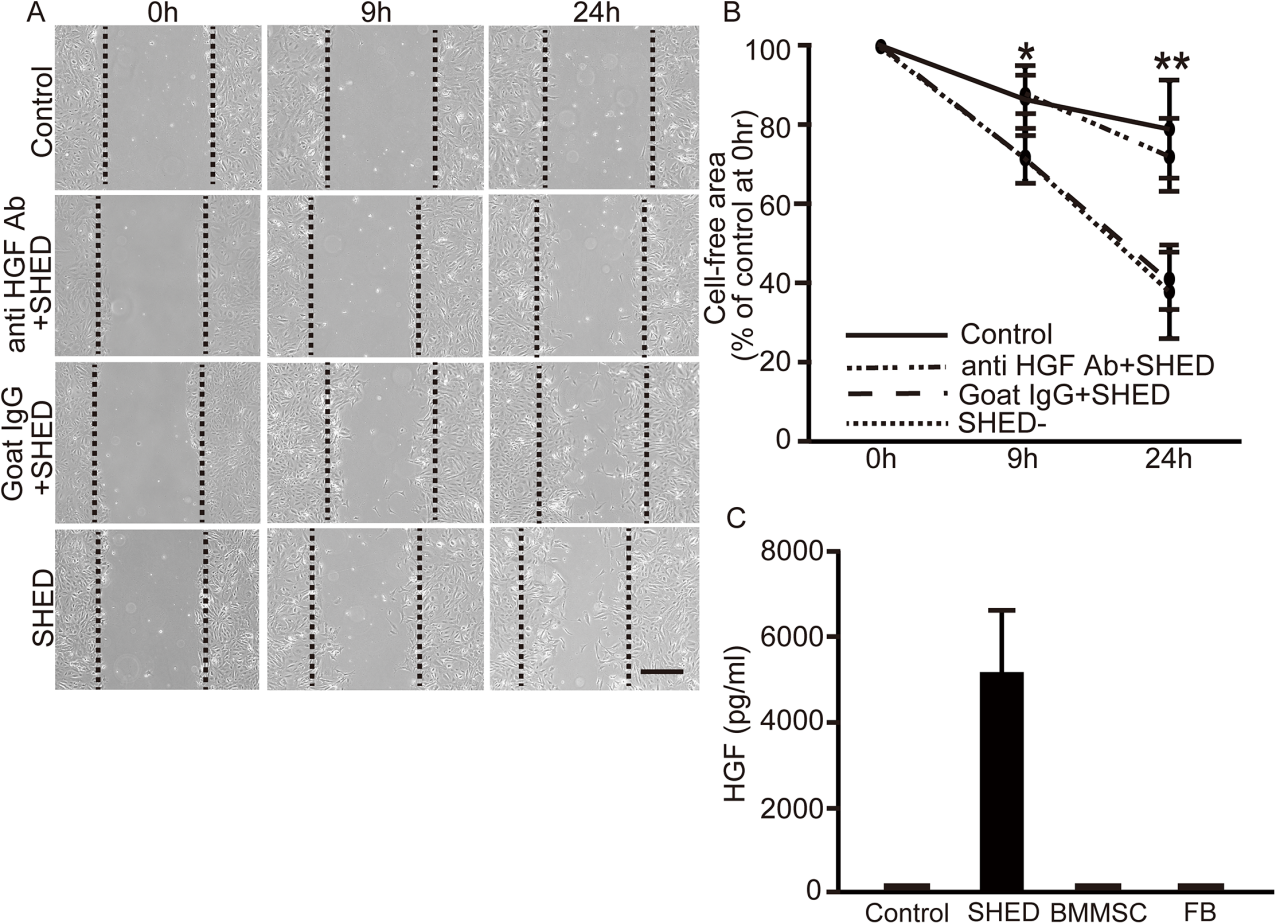
Scratch wound healing assays of TEC. Repair of TEC is accelerated by SHED-CM and anti-HGF antibody blunted the effects in scratch wound-healing assays. Injured TEC monolayers were incubated with SHED-CM (SHED), SHED-CM with anti HGF antibody (anti HGF Ab+ SHED), SHED-CM with normal goat IgG (Goat IgG+ SHED) or control medium (Control). (A) Representative images of the progression of wound closure at 0 h, 9 h, and 24 h (original magnification, x5 Scale bar: 300 μmm), black dotted lines indicate initial injury. Cells growing over the lines represent injury closure. Each assay was performed in triplicate and experiments were repeated five times. (B) Evaluation of the scratch wound healing assay. The percentage of cell-free area at indicated time points compared with the area at the start of experiment (0 h) was determined. Values are presented as mean ±SD; SHED group (n = 5); anti HGF antibody group (n = 5); normal goat IgG group (n = 5); control group (n = 5).* *P*<0.01 SHED vs. control, normal goat IgG vs. control, SHED vs. anti HGF antibody, and normal goat IgG vs. anti HGF antibody. **P<0.001 SHED vs. control, normal goat IgG vs. control, SHED vs. anti HGF antibody, and normal goat IgG vs. anti HGF antibody. (C) Expression of HGF in conditioned media from BMMSC, FB, and SHED at 48 h. Each assay was performed in triplicate and experiments were repeated three times. Values are presented as mean ±SD.

To study the potential of SHED-CM to promote cell proliferation and migration, scratch wound healing assays were performed. Repair of injured TEC was accelerated by SHED-CM at 9 h and 24 h, indicating that SHED secreted factors promoted cell proliferation and migration (Fig 6A and 6B). In order to examine the role of HGF in the SHED-CM on TEC proliferation and migration, anti-HGF neutralizing antibody was used. Treatment with anti-HGF antibody blunted the effect of SHED-CM, while normal goat IgG showed no effect.

#### HGF expression in conditioned media

Since HGF is known to play a role in the regeneration and protection of the kidney, we measured HGF secreted from BMMSC, FB, and SHED. SHED produced higher levels of HGF than BMMSC, FB, and the control (Fig 6C).

## Discussion

SHED, a type of MSC, have been shown to exert curative effects in various animal models, including bone defect, skin ulcer, cord injury and neonatal hypoxia-ischemia [15–18]. The therapeutic function of SHED has been attributed to anti-inflammatory effects, anti-apoptosis effects, and paracrine effects. We found that subrenal capsular injection of SHED, but not BMMSC, showed renoprotective effects in a mice model of ischemic AKI. To the best of our knowledge this study is the first to successfully demonstrate that administration of SHED reduces inflammatory change and tissue injury and improves renal function in AKI.

Previous studies have demonstrated that various types of MSC improve renal function [34–36]. However, the mechanisms remain contentious. Several studies indicated that administered MSC exert their therapeutic effect by replacing damaged cells with differentiated stem cells [37, 38]. Nevertheless, differentiation and incorporation of MSC into damaged tissues is observed at widely varying rates of approximately 0.1 to 20%, and the contribution of this process to improve renal function and regeneration has not been conclusive [39–41]. Recent studies have demonstrated that factors secreted by administered stem cells protect tissues against injury through paracrine mechanisms [42–45]. MSCs secrete many factor, these include a variety of growth factors, cytokines and chemokines. In addition to these factors, more recent studies demonstrated that MSCs also secrete extracellular vesicles which consist of exosomes and shedding vesicles. These are collectively defined as microvesicles (MVs) and their therapeutic effects for AKI have been reported [46–49]. MVs comprise a variety of substance (such as receptors, adhesion molecules, lipids of the cell membrane, mRNA, microRNA, DNA). MVs operate as a cargo for delivering cellular constituent to other cell, accordingly alterations in the behavior and phenotype of recipient cells [50]. In this study, curative effects of BMMSC were not observed. We also reported that BMMSC were not effective while adipose derived MSC effectively attenuated kidney damage [23, 51]. The discrepancy between SHED and BMMSC may be attributed to different microRNA cargos.

Previously, we have shown that human adipose tissue-derived stromal cells (ASC) attenuated folic acid-induced AKI through the secretion of HGF. ASC, administered into the subrenal capsule, remained at the site of injection without infiltration into the kidney tissue at day 14 [23]. In the present study, administered SHED were also localized at the subrenal capsule, and we could not identify any cells that had infiltrated into the kidney. Therefore, this is one of the reasons which we conclude that SHED protected the kidney from ischemic injury by a paracrine effect.

Regarding the mechanism underlying SHED capabilities, our results suggest SHED possess anti-inflammatory activity. Ischemic AKI represents an inflammatory disease [52]. The initial process of IRI inflammation is induced by endothelial cell injury followed by pro-inflammatory chemokine secretion from tubular epithelial cells, leading to pro-inflammatory leukocyte infiltration in the kidney [52]. Tubular epithelial cells produce pro-inflammatory chemokines, such as MIP-2 and MCP-1 [52–54]. MCP-1/CCR2 (chemokine receptor of MCP-1) signaling mediates macrophage infiltration [55, 56]. 7ND, an MCP-1 antagonist, has been shown to attenuate tubular damage, decrease the number of inflammatory cells, and reduce organ fibrosis [57, 58]. Moreover, IL-1β is secreted by macrophages and participates in the inflammation process. In the present study, the number of inflammatory cells including neutrophils and macrophages was significantly reduced and the levels of MCP-1, MIP-2, and IL-1β were significantly lower in the SHED treatment group than in the control group *in vivo*. Additionally, SHED-CM reduced TEC MCP-1 expression *in vitro*. These results indicate that SHED possess an anti-inflammatory ability and that SHED secreted factors exert a renoprotective effect by paracrine action.

Our study also suggests that SHED promote wound healing; in a scratch wound healing assay, SHED-CM accelerated repair of injured TEC. These data indicate that SHED secreted factors promote cell proliferation and migration by paracrine mechanisms. It has been shown that HGF enhances the proliferation and migration of tubular epithelial cells and promotes repair of injured tubular cells [23, 59, 60]. In this study, adding an anti-HGF antibody to the SHED-CM resulted in significantly reduced wound healing. These results suggest that SHED produced and secreted HGF, which promote the restoration of TEC.

In this study, SHED were administered locally to the subrenal capsule of the kidney. There is advantage of subrenal capsule injection. Compared with subrenal capsule injection, most of cells are trapped in the lungs after intravenous injection. Furthermore, it is thought that subrenal capsule injection has little effect for the body while the effect remains only at the local site. In addition, subrenal capsule injection may need fewer cells. In the clinical setting, subrenal capsule injection will be feasible for patients who undergo renal transplantation.

There are limitations in the present study. In this study, human cells were injected into a mice model of AKI. Our results showed that the host vs. graft reaction was observed on days 7 and 14 after injection of SHED or BMMSC. Therefore, it was difficult to determine the differentiation capacity of SHED or BMMSC into renal cells. In addition, it may be difficult to compare our results with those in other studies because the route of administration, number of cell and the condition of the model were different. Finally, the molecular mechanisms underlying these effects largely remain to be elucidated. Further studies will be required to explore the factors secreted from SHED, which exert renoprotective activity in AKI.

In conclusion, administration of SHED attenuated tissue injury in ischemic AKI. Our study suggests that SHED exerted anti-inflammatory activity during the early stage of inflammation and promoted proliferation and migration of tubular epithelial cells through a paracrine mechanism. SHED might provide a novel stem cell resource, which could be applied for the treatment of ischemic kidney injury.

## Supporting Information

S1 Fig Tracking of administered BMMSC. H&E staining in subrenal capsule. (A) Representative micrographs of administered SHED in subrenal capsule using anti-human lamin A/C (arrows, original magnification, x100 Scale bar: 200 μm). BMMSC group, day2 (n = 4), day7 (n = 4), day14 (n = 4); control group, day2 (n = 4), day7 (n = 4), day 14 (n = 4). (B) Representative micrographs of administered SHED in subrenal capsule stained with H&E (arrow heads: inflammatory cells, original magnification, x100 Scale bar: 200 μmm). SHED group, day2 (n = 4), day7 (n = 4), day14 (n = 4); control group, day2 (n = 4), day7 (n = 4), day 14 (n = 4). (C) Representative micrographs of administered BMMSC in subrenal capsule stained with H&E (arrow heads: inflammatory cells, original magnification, x100 Scale bar: 200 μmm). SHED group, day2 (n = 4), day7 (n = 4), day14 (n = 4); control group, day2 (n = 4), day7 (n = 4), day 14 (n = 4).
(TIF)

S2 Fig Immunofluorescence staining of neutrophils, macrophages and T cells in subrenal capsule after SHED administration. (A) Representative immunofluorescence staining of neutrophils using anti-Ly-6B (arrows, original magnification, x200 Scale bar: 200 μmm). (B) Representative immunofluorescence staining using ratIgG2a (negative control for Ly-6B) (original magnification, x200 Scale bar: 200 μmm). (C) Representative immunofluorescence staining of macrophages using anti-F4/80 (original magnification, x200 Scale bar: 200 μmm). (D) Representative immunofluorescence staining using rat IgG2b (negative control for F4/80) (original magnification, x200 Scale bar: 200 μmm). (E) Representative immunofluorescence staining of T cells using anti-CD3 (original magnification, x200 Scale bar: 200 μmm). (F) Representative immunofluorescence staining using rabbit IgG (negative control for CD3) (original magnification, x200 Scale bar: 200 μmm). SHED group, day2 (n = 4), day7 (n = 4), day14 (n = 4); control group, day2 (n = 4), day7 (n = 4), day 14 (n = 4).
(TIF)

S3 Fig Immunofluorescence staining of neutrophils, macrophages and T cells in subrenal capsule after BMMSC administration. (A) Representative immunofluorescence staining of neutrophils using anti-Ly-6B (arrows, original magnification, x200 Scale bar: 200 μmm). (B) Representative immunofluorescence staining using ratIgG2a (negative control for Ly-6B) (original magnification, x200 Scale bar: 200 μmm). (C) Representative immunofluorescence staining of macrophages using anti-F4/80 (original magnification, x200 Scale bar: 200 μmm). (D) Representative immunofluorescence staining using rat IgG2b (negative control for F4/80) (original magnification, x200 Scale bar: 200 μmm). (E) Representative immunofluorescence staining of T cells using anti-CD3 (original magnification, x200 Scale bar: 200 μmm). (F) Representative immunofluorescence staining using rabbit IgG (negative control for CD3) (original magnification, x200 Scale bar: 200 μmm). BMMSC group, day2 (n = 4), day7 (n = 4), day14 (n = 4); control group, day2 (n = 4), day7 (n = 4), day 14 (n = 4).
(TIF)

S4 Fig Negative Control staining of neutrophils, macrophages. (A) Representative immunofluorescence staining using ratIgG2a (negative control for Ly-6B) (original magnification, x200 Scale bar: 200 μmm). (B) Representative immunofluorescence staining rat IgG2b (negative control for F4/80) (original magnification, x400 Scale bar: 100 μmm). SHED group, day1 (n = 9), day2 (n = 8), day7 (n = 8); control group, day1 (n = 9), day2 (n = 7), day 7 (n = 7).
(TIF)

## Abbreviations

AKI: Acute kidney injury
SHED: Stem cells from human exfoliated deciduous teeth
IRI: Ischemia-reperfusion injury
PBS: Phosphate-buffered saline
BUN: Blood urea nitrogen
MIP-2: Macrophage inflammatory protein
IL-1β: Interleukin-1 β
MCP-1: Monocyte chemotactic protein-1
H_2_O_2_: Hydrogen peroxide
TEC: Tubular epithelial cells
MSC: Mesenchymal stem cells
BMMSC: Bone marrow–derived mesenchymal stem cells
DMEM: Dulbecco's modified Eagle medium
SHED-CM: Conditioned medium from stem cells from human exfoliated deciduous teeth
HGF: Hepatocyte growth factor
sCr: Serum creatinine
PAS: Periodic acid-Schiff staining
ATN: Acute tubular necrosis score
ELISA: Enzyme-linked immunosorbent assay
FB: Human skin-fibroblasts
ASC: Adipose tissue-derived stromal cells
Ab: Antibody
MVs: Microvesicles
