# Correction: Therapeutic Potential of Stem Cells from Human Exfoliated Deciduous Teeth in Models of Acute Kidney Injury

**DOI:** 10.1371/journal.pone.0143561

**Published:** 2015-11-16

**Authors:** Yuka Hattori, Hangsoo Kim, Naotake Tsuboi, Akihito Yamamoto, Shinichi Akiyama, Yiqin Shi, Takayuki Katsuno, Tomoki Kosugi, Minoru Ueda, Seiichi Matsuo, Shoichi Maruyama

There are errors in the Funding section. The correct funding information is as follows: A research grant from Aichi Kidney Foundation, YH. The COE for education and research of Micro-Nano Mechatronics Nagoya University Global COE Program, YH KH. Funding was also received from Kyowa Hakko Kirin Co., Ltd; Otsuka Pharmaceutical Co.,Ltd; Dainippon Sumitomo Pharma Co., Ltd; and MOCHIDA PHARMACEUTICAL CO. LTD. The funders had no role in study design, data collection and analysis, decision to publish, or preparation of the manuscript.
