# Supplementary figures and images for: Therapeutic Potential of Stem Cells from Human Exfoliated Deciduous Teeth in Models of Acute Kidney Injury
Source: PLoS One. 2015 Oct 28;10(10):e0140121. doi: 10.1371/journal.pone.0140121 (PMC4625005; doi:10.1371/journal.pone.0140121)

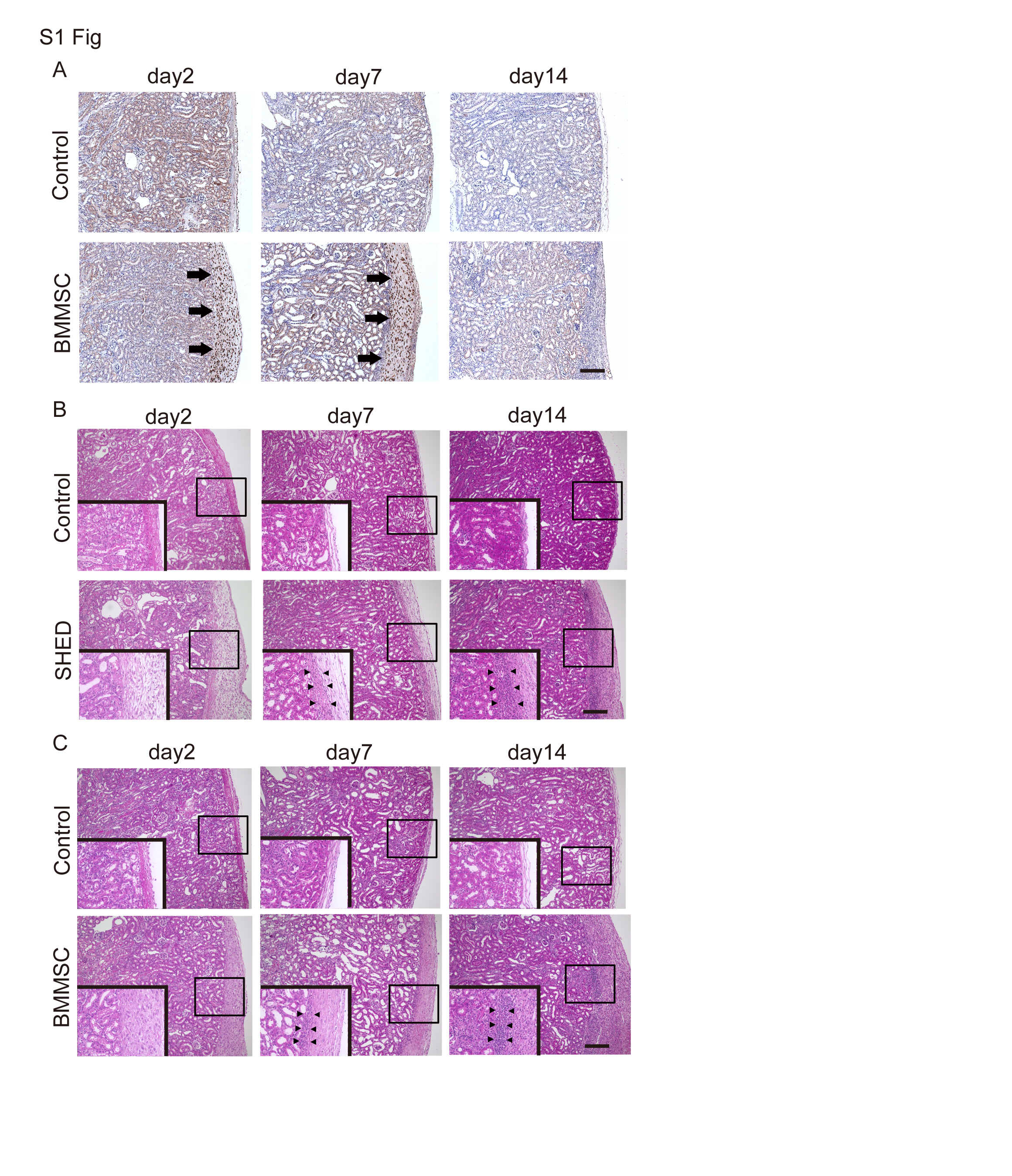

Supplement: S1 Fig — (A) Representative micrographs of administered SHED in subrenal capsule using anti-human lamin A/C (arrows, original magnification, x100 Scale bar: 200 μm). BMMSC group, day2 (n = 4), day7 (n = 4), day14 (n = 4); control group, day2 (n = 4), day7 (n = 4), day 14 (n = 4). (B) Representative micrographs of administered SHED in subrenal capsule stained with H&E (arrow heads: inflammatory cells, original magnification, x100 Scale bar: 200 μmm). SHED group, day2 (n = 4), day7 (n = 4), day14 (n = 4); control group, day2 (n = 4), day7 (n = 4), day 14 (n = 4). (C) Representative micrographs of administered BMMSC in subrenal capsule stained with H&E (arrow heads: inflammatory cells, original magnification, x100 Scale bar: 200 μmm). SHED group, day2 (n = 4), day7 (n = 4), day14 (n = 4); control group, day2 (n = 4), day7 (n = 4), day 14 (n = 4). (TIF) [file pone.0140121.s001.tif]

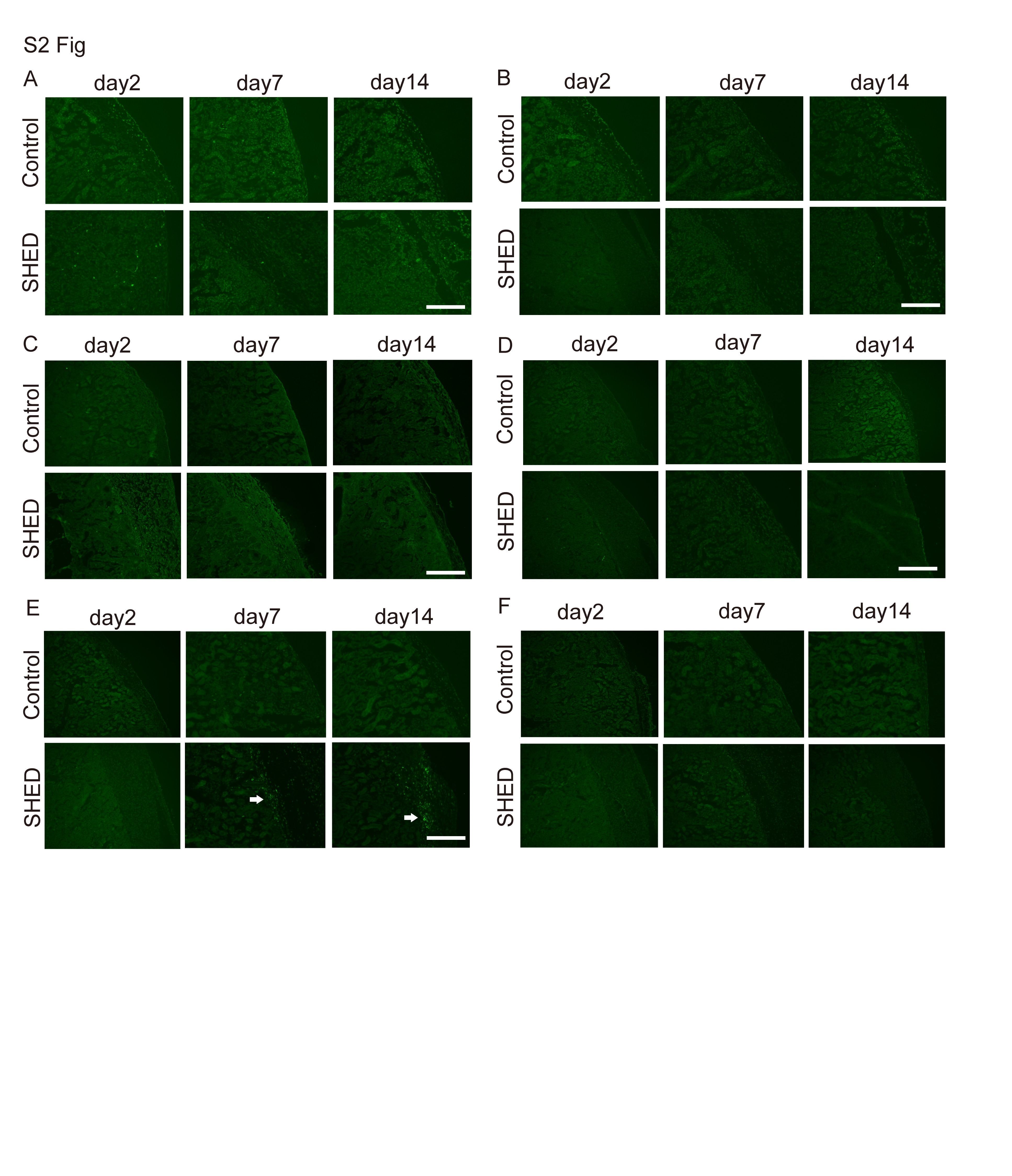

Supplement: S2 Fig — (A) Representative immunofluorescence staining of neutrophils using anti-Ly-6B (arrows, original magnification, x200 Scale bar: 200 μmm). (B) Representative immunofluorescence staining using ratIgG2a (negative control for Ly-6B) (original magnification, x200 Scale bar: 200 μmm). (C) Representative immunofluorescence staining of macrophages using anti-F4/80 (original magnification, x200 Scale bar: 200 μmm). (D) Representative immunofluorescence staining using rat IgG2b (negative control for F4/80) (original magnification, x200 Scale bar: 200 μmm). (E) Representative immunofluorescence staining of T cells using anti-CD3 (original magnification, x200 Scale bar: 200 μmm). (F) Representative immunofluorescence staining using rabbit IgG (negative control for CD3) (original magnification, x200 Scale bar: 200 μmm). SHED group, day2 (n = 4), day7 (n = 4), day14 (n = 4); control group, day2 (n = 4), day7 (n = 4), day 14 (n = 4). (TIF) [file pone.0140121.s002.tif]

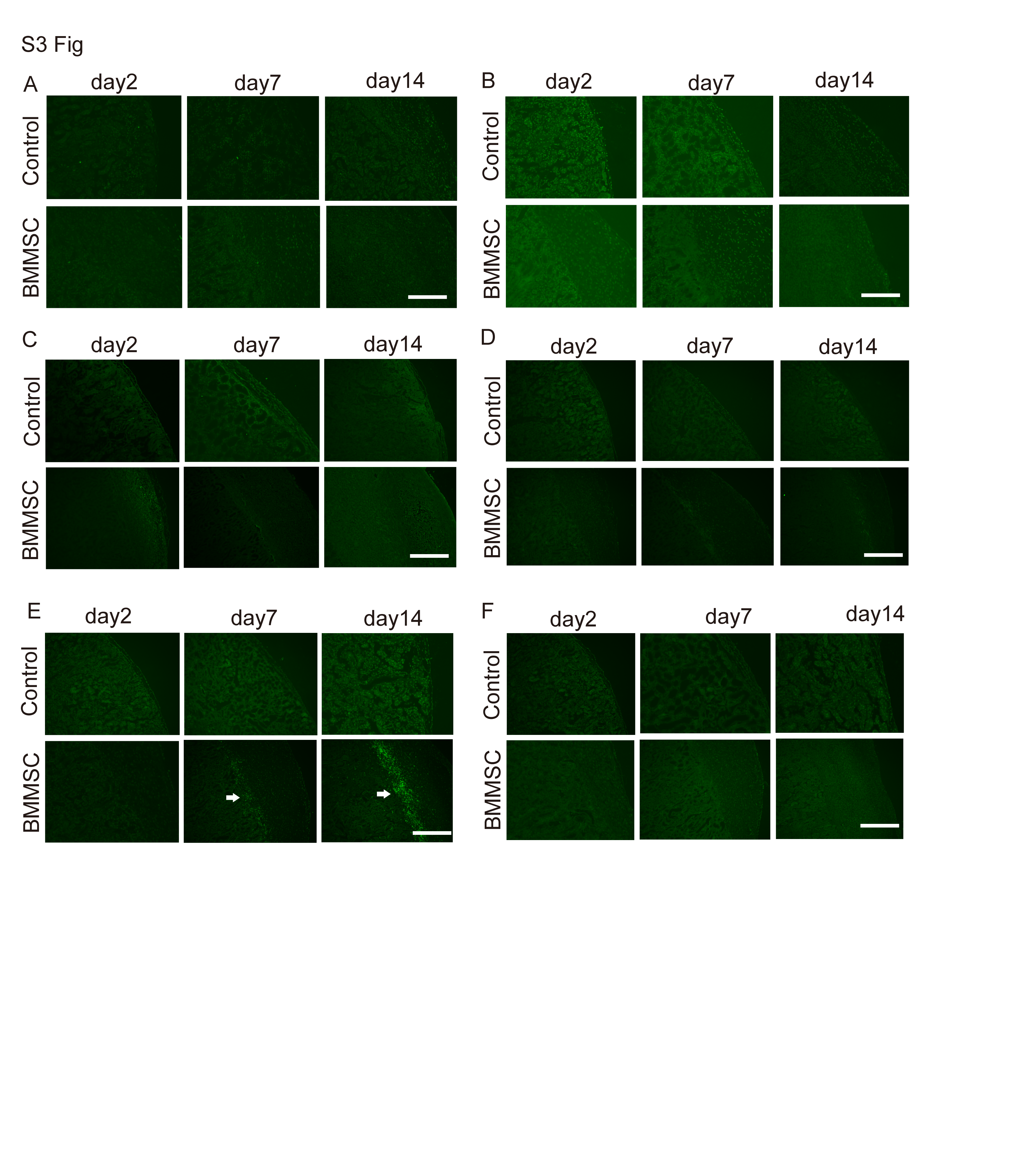

Supplement: S3 Fig — (A) Representative immunofluorescence staining of neutrophils using anti-Ly-6B (arrows, original magnification, x200 Scale bar: 200 μmm). (B) Representative immunofluorescence staining using ratIgG2a (negative control for Ly-6B) (original magnification, x200 Scale bar: 200 μmm). (C) Representative immunofluorescence staining of macrophages using anti-F4/80 (original magnification, x200 Scale bar: 200 μmm). (D) Representative immunofluorescence staining using rat IgG2b (negative control for F4/80) (original magnification, x200 Scale bar: 200 μmm). (E) Representative immunofluorescence staining of T cells using anti-CD3 (original magnification, x200 Scale bar: 200 μmm). (F) Representative immunofluorescence staining using rabbit IgG (negative control for CD3) (original magnification, x200 Scale bar: 200 μmm). BMMSC group, day2 (n = 4), day7 (n = 4), day14 (n = 4); control group, day2 (n = 4), day7 (n = 4), day 14 (n = 4). (TIF) [file pone.0140121.s003.tif]

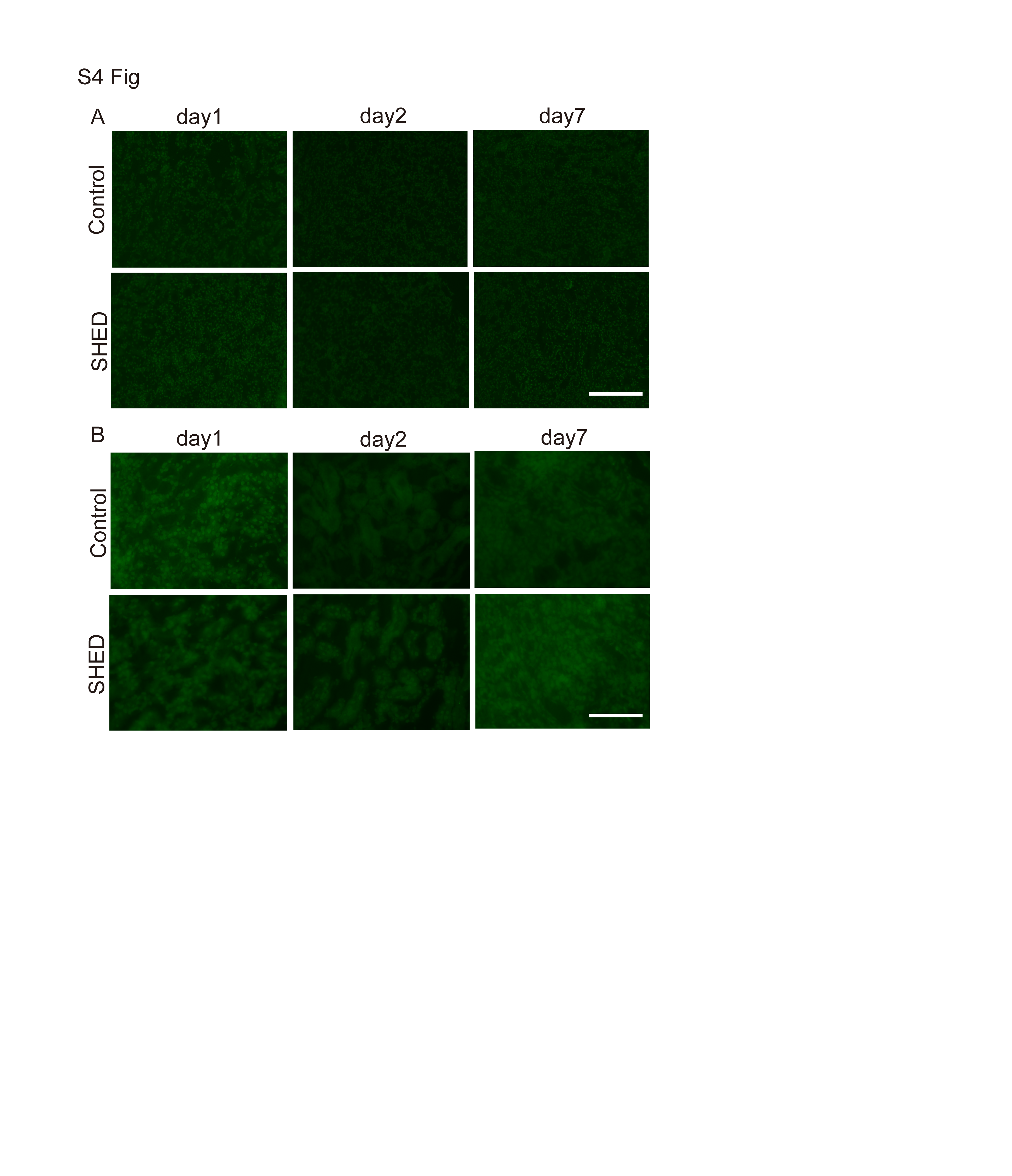

Supplement: S4 Fig — (A) Representative immunofluorescence staining using ratIgG2a (negative control for Ly-6B) (original magnification, x200 Scale bar: 200 μmm). (B) Representative immunofluorescence staining rat IgG2b (negative control for F4/80) (original magnification, x400 Scale bar: 100 μmm). SHED group, day1 (n = 9), day2 (n = 8), day7 (n = 8); control group, day1 (n = 9), day2 (n = 7), day 7 (n = 7). (TIF) [file pone.0140121.s004.tif]
